# Spermine-Salicylic Acid Interplay Restrains Salt Toxicity in Wheat (*Triticum aestivum* L.)

**DOI:** 10.3390/plants12020352

**Published:** 2023-01-12

**Authors:** Neveen B. Talaat, Alaa M. A. Hanafy

**Affiliations:** Department of Plant Physiology, Faculty of Agriculture, Cairo University, Giza 12613, Egypt

**Keywords:** antioxidant system, Calvin cycle, root H^+^-pump, salicylic acid, salinity, spermine, *Triticum aestivum*

## Abstract

Spermine (SPM) and salicylic acid (SA) are plant growth regulators, eliciting specific responses against salt toxicity. In this study, the potential role of 30 mgL^−1^ SPM and/or 100 mgL^−1^ SA in preventing salt damage was investigated. Wheat plants were grown under non-saline or saline conditions (6.0 and 12.0 dS m^−1^) with and without SA and/or SPM foliar applications. Exogenously applied SA and/or SPM alleviated the inhibition of plant growth and productivity under saline conditions by increasing Calvin cycle enzyme activity. Foliage applications also improved ascorbate peroxidase, monodehydroascorbate reductase, dehydroascorbate reductase, and glutathione reductase activities, which effectively scavenged hydrogen peroxide and superoxide radicals in stressed plants. Furthermore, foliar treatments increased antioxidants such as ascorbate and glutathione, which effectively detoxified reactive oxygen species (ROS). Exogenous applications also increased N, P, and K^+^ acquisition, roots’ ATP content, and H^+^-pump activity, accompanied by significantly lower Na^+^ accumulation in stressed plants. Under saline environments, exogenous SA and/or SPM applications raised endogenous SA and SPM levels. Co-application of SA and SPM gave the best response. The newly discovered data suggest that the increased activities of Calvin cycle enzymes, root H^+^-pump, and antioxidant defense machinery in treated plants are a mechanism for salt tolerance. Therefore, combining the use of SA and SPM can be a superior method for reducing salt toxicity in sustainable agricultural systems.

## 1. Introduction 

Salt stress is one of the most significant environmental challenges resulting in significant crop losses. It is expected that 50% of arable land will be under saline environments by 2050 due to environmental pollution, lack of fresh water, improper irrigation methods, and other factors [1]. It is a complex abiotic stress that causes nutritional imbalances, particular ionic effects, and osmotic stress. For defense, plants minimize the entry of salt ions into the cytoplasm and/or enhance the synthesis of osmolytes, antioxidants, and phytohormones [2,3,4]. Salt stress also induces the over-production of reactive oxygen species (ROS) causing oxidative stress that impairs cellular homeostasis. ROS, as strong oxidants, can result in lipid peroxidation, protein denaturation, DNA mutation, and finally cell death [5]. Plants have integrated ROS detoxification machinery, which includes antioxidant enzymes [superoxide dismutase (SOD), catalase (CAT), ascorbate peroxidase (APX), glutathione reductase (GR), monodehydroascorbate reductase (MDHAR), and dehydroascorbate (DHAR)] as well as non-enzymatic antioxidants [ascorbic acid (AsA), reduced glutathione (GSH), α-tocopherol, carotenoids, phenols, and flavonoids] [6,7]. Ionic status within the plant cell is also critical for salt tolerance because excessive salt ions in the cytoplasm reduce nutrient absorption, inhibit photosynthesis, and affect water transport, thus hindering plant growth and productivity [4,8]. Plants adapt to salt stress and mitigate its adverse effects through various methods and physiological mechanisms. For example, excess sodium ions are compartmentalized into vacuoles and/or transported by apoplast pathways. The Na^+^/H^+^-antiporter mediates the sequestration of sodium in the vacuole, which is facilities by an action of the vacuolar H^+^-ATPase and H^+^-PPase [9]. It has been reported that sodium causes an increase in vacuolar H^+^-ATPase activity in salt-stressed plants [10,11]. Furthermore, salt stress has an impact on photosynthesis activity [4,8]. The Calvin cycle is the main pathway by which plants fix carbon. Salinity inhibits the activity of Calvin cycle enzymes, such as ribulose diphosphate carboxylase/oxygenase (Rubisco), thereby altering the photosynthetic rate [8]. 

Salicylic acid (SA, 2-hydroxybenzoic acid) is known as a plant growth regulator that plays important roles under different environmental stresses [6]. Evidence shows that SA might have a critical role in regulating photosynthetic processes in plants subjected to salt stress [12,13,14]. Furthermore, SA application improved plant growth and development under saline conditions by enhancing the mineral element content, antioxidant defense machinery, methylglyoxal detoxification system, protein synthesis, and ROS detoxification [4,6,7,13,15]. 

Spermine (SPM, tetraamine) is also considered a plant growth regulator and a secondary messenger in signaling pathways involved in abiotic stress tolerance [16,17,18]. Exogenously applied SPM prevented salt damage by actively contributing to osmotic adjustment, free radical scavenging, photosynthetic efficiency, maintaining cationic-anionic stability, lowering ethylene production, enhancing protein content, modifying the levels of endogenous phytohormones, and inducing organic solute accumulation [18,19,20,21]. SPM interacts with other phytohormones such as salicylic acid, abscisic acid, brassinosteroids, and ethylene, to coordinate the reactions necessary for developing stress tolerance [22]. 

Wheat (*Triticum aestivum* L.) is the most important cereal crop, and salinity levels of 6–8 dS m^−1^ cause declines in wheat yield [3,23]. Improving its adaptation to saline conditions is thought to be the most effective economical approach. Some studies have looked into the effect of a single application of SA or SPM on plants exposed to salt stress, but no one has looked into the impact of their combined treatment on wheat salt tolerance. To fill this gap, as a first investigation, we conducted this study to assess the impact of SA and SPM combined treatment on the antioxidant defense machinery, Calvin cycle enzymes activity, and root H^+^-pump activity of wheat plants grown under saline conditions. The purpose of this study was to verify the hypothesis that the dual application of SA and SPM could ameliorate the damaging effects of salt-induced oxidative stress and nutrient imbalance to improve wheat salt tolerance. The positive effects of SA and/or SPM foliar applications were investigated by measuring plant growth and productivity parameters, Calvin cycle enzymes [ribulose diphosphate carboxylase/oxygenase (Rubisco), fructose 1,6-bisphosphatase (FBPase), glyceraldehyde 3-phosphate dehydrogenase (GAPDH), and fructose 1,6-bisphosphate aldolase (FBA)] activity, nutrient (N, P, K^+^, Na^+^) acquisition, ATP content, H^+^-pump activity, reactive oxygen species (H_2_O_2_, O_2_^•−^) content, antioxidant enzymes (APX, MDHAR, DHAR, GR) activity, antioxidant molecules (GSH, AsA) content, as well as the endogenous SA and SPM concentration of wheat (*Triticum aestivum* L. cv. Shandawel 1) plants subjected to non-saline and saline (6.0 and 12.0 dS m^−1^) conditions. This research provides novel insights into the mechanisms of SA- and SPM-mediated amelioration of salt stress on wheat. 

## 2. Results 

### 2.1. Foliage Applications of SA and/or SPM Promote Wheat Growth and Productivity under Salt Stress

**Figure 1:**
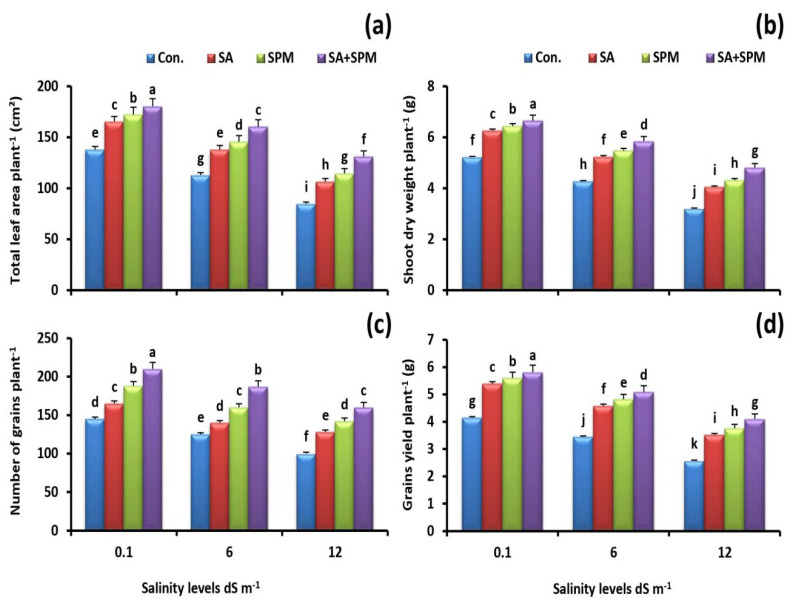
Effect of salicylic acid (SA) Spermine (SPM) foliage applications on growth and yield attributes (**a**) total leaf area plant-1, (**b**) shoot dry weight plant-1, and (**c**) number of grains plant-1 and (**d**) grains yield plant-1 of wheat plants exposed to non-saline and saline (6.0 and 12.0 dS m^−1^) conditions. The results showed the mean ± SE of four replicates. Different letters indicate significant differences at (*p* < 0.05) level according to Duncan’s test.

Saline conditions resulted in a sharp decline in the growth and yield of wheat plants in terms of total leaf area, dry weight of shoot, grain number, and grain yield. On the contrary, foliage applications of SA and/or SPM significantly alleviated the salt toxicity and attenuated the inhibitory impact of salt on these parameters (Figure 1a–d). The combined treatment of SA and SPM produced the best results. When compared to values of untreated plants at a salinity level of 12.0 dS m^−1^, combined treatment significantly (*p* < 0.05) increased the leaf area by 55.0%, shoot dry weight by 49.8%, grain number by 60.0%, and grain yield by 59.5%. 

### 2.2. Exogenously Applied SA and/or SPM Enhance the Activity of Calvin Cycle Enzymes under Saline Conditions

**Figure 2:**
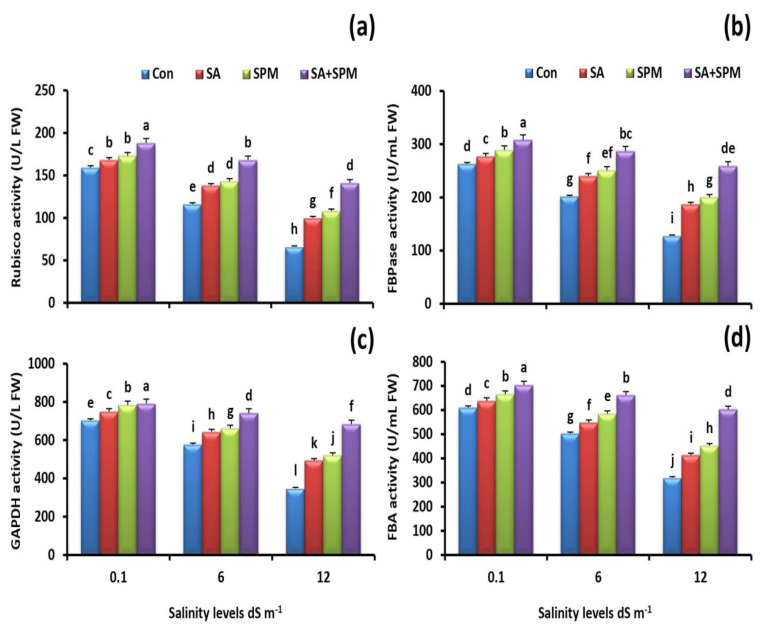
Effect of salicylic acid (SA) and/or spermine (SPM) foliage applications on the activity of (**a**) ribulose diphosphate carboxylase/oxygenase (Rubisco), (**b**) fructose 1,6-bisphosphatase (FBPase), (**c**) glyceraldehyde-3-phosphate dehydrogenase (GAPDH), and (**d**) fructose 1,6-bisphosphate aldolase (FBA) in leaves of wheat plants exposed to non-saline and saline (6.0 and 12.0 dS m^−1^) conditions. The results showed the mean ± SE of four replicates. Different letters indicate significant differences at (*p* < 0.05) level according to Duncan’s test.

As shown in Figure 2a–d, salt-stressed wheat plants were associated with considerably lower Rubisco, FBPase, GAPDH, and FBA activities than those of the unstressed plants. By contrast, foliage applications of SA and/or SPM significantly ameliorated salt injuries and increased the activity of these enzymes. The combination of SA and SPM treatment revealed the greatest impact. When compared to readings of untreated plants at a salinity level of 12.0 dS m^−1^, combined treatment reduced the negative effects of salt and significantly (*p* < 0.05) enhanced the activity of Rubisco by 113.6%, FBPase by 102.3%, GAPDH by 96.6%, and FBA by 88.4%.

### 2.3. Spraying of SA and/or SPM Enhance Nutrient Acquisition in Salt-Stressed Wheat Plants

**Figure 3:**
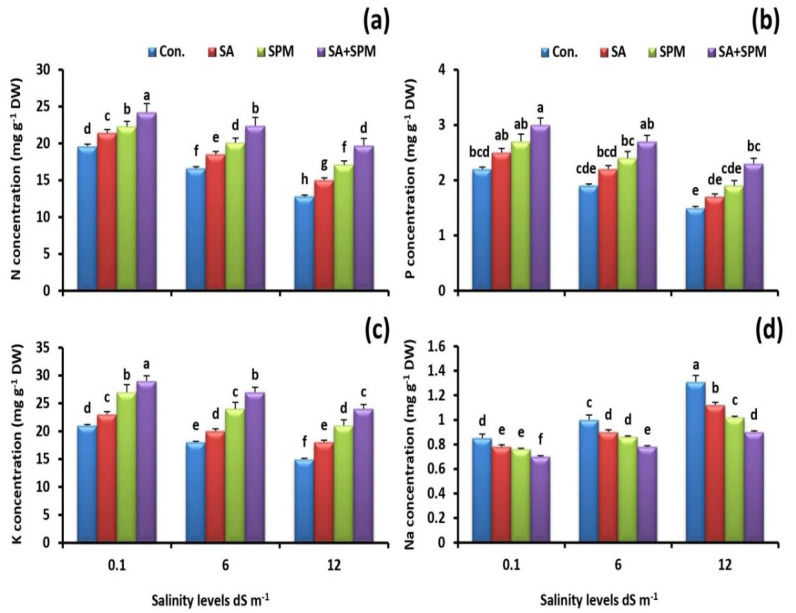
Effect of salicylic acid (SA) and/or spermine (SPM) foliage applications on the concentration of (**a**) nitrogen (N), (**b**) phosphorus (P), (**c**) potassium (K), and (**d**) sodium (Na) in grains of wheat plants exposed to non-saline and saline (6.0 and 12.0 dS m^−1^) conditions. The results showed the mean ± SE of four replicates. Different letters indicate significant differences at (*p* < 0.05) level according to Duncan’s test.

Alteration of ion homeostasis was assessed in grains of wheat plants. The concentrations of N, P, and K^+^ were negatively affected under saline conditions; moreover, this effect was most marked at high salinity levels. Interestingly, exogenous SA and/or SPM treatments alleviated the detrimental injuries of salt stress on the mineral acquisition by improving N, P, and K^+^ acquisition (Figure 3a–c) as well as reducing Na^+^ accumulation (Figure 3d). The best results were obtained by combining SA and SPM. When compared to untreated plants at a salinity level of 12.0 dS m^−1^, SA and SPM combined treatment significantly (*p* < 0.05) increased N, P, and K^+^ concentrations by 53.9%, 53.3%, and 60.0%, respectively, while significantly decreasing Na^+^ level by 31.3% in grains of wheat plants. 

### 2.4. Foliar Applications of SA and/or SPM Improve Roots’ ATP Content and H^+^-Pump Activity in Salt-Stressed Wheat Plants 

**Figure 4:**
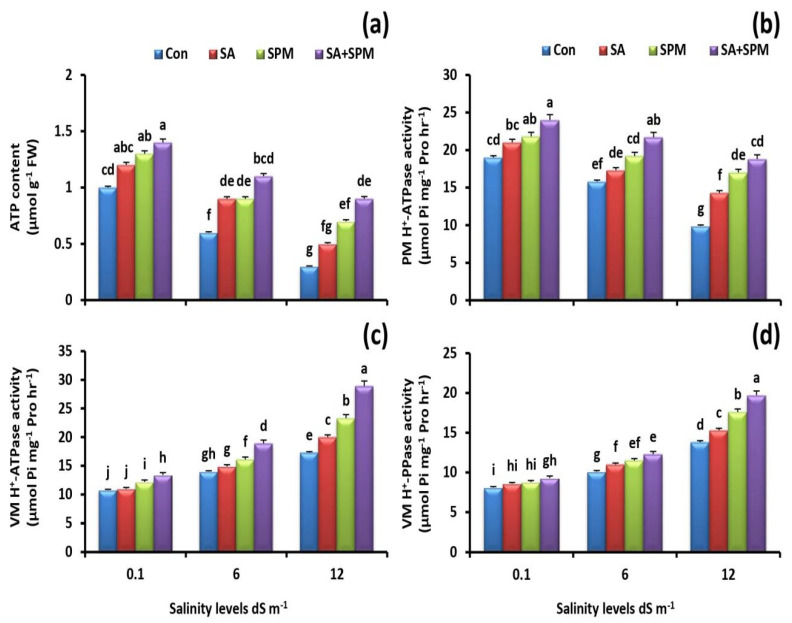
Effect of salicylic acid (SA) and/or spermine (SPM) foliage applications on the (**a**) ATP content, (**b**) plasma membrane (PM) H^+^-ATPase activity, (**c**) vacuole membrane (VM) H^+^-ATPase activity, and (**d**) vacuole membrane (VM) H^+^-PPase activity in roots of wheat plants exposed to non-saline and saline (6.0 and 12.0 dS m^−1^) conditions. The results showed the mean ± SE of four replicates. Different letters indicate significant differences at (*p* < 0.05) level according to Duncan’s test.

Salinity markedly decreased the roots’ ATP content and PM H^+^-ATPase activity, while SA and/or SPM treatments ameliorated the inhibitory impact induced by salt stress and significantly enhanced their levels (Figure 4a,b). When compared to untreated plants, co-application of SA and SPM improved ATP content and PM H^+^-ATPase activity by 200% and 89.9%, respectively, in the roots of wheat plants grown under 12.0 dS m^−1^ salinity level.

It is interesting to note that salt stress treatments rose the activity of VM H^+^-ATPase and VM H^+^-PPase. In addition, SA and/or SPM treatments under saline conditions further boosted their activities (Figure 4c,d). The SA and SPM co-application detected the most actions. In comparison to untreated plants, combined treatment under 12.0 dS m^−1^ salinity level significantly (*p* < 0.05) increased the activity of VM H^+^-ATPase by 67.1% and that of VM H^+^-PPase by 42.8% in the roots of wheat plants. 

### 2.5. Foliage Applications of SA and/or SPM Detoxify ROS Molecules under Salt Stress

**Figure 5:**
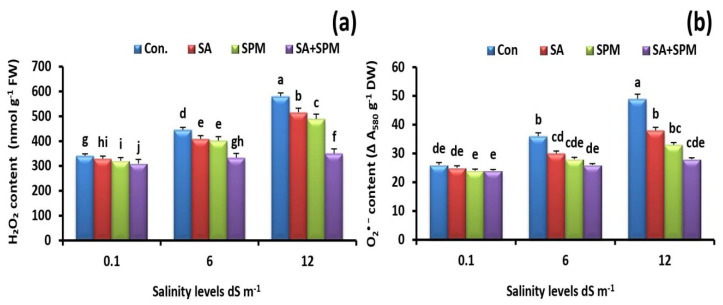
Effect of salicylic acid (SA) and/or spermine (SPM) foliage applications on the content of (**a**) hydrogen peroxide (H_2_O_2_) and (**b**) superoxide (O_2_^•−^) in leaves of wheat plants exposed to non-saline and saline (6.0 and 12.0 dS m^−1^) conditions. The results showed the mean ± SE of four replicates. Different letters indicate significant differences at (*p* < 0.05) level according to Duncan’s test.

To investigate if SA and/or SPM treatments alleviate salt stress-induced oxidative stress, the generation of H_2_O_2_ and O_2_^•−^ in wheat leaves was detected. Salt stress caused a considerable increase in their concentrations. Conversely, exogenous SA and/or SPM applications significantly (*p* < 0.05) mitigated this adverse impact and restored their production to a similar level as in unstressed plants (Figure 5a,b). The best impact was detected with SA and SPM combined treatment. It neutralized salt-generated toxic effects by reducing the H_2_O_2_ and O_2_^•−^ content in leaves of wheat plants by 39.7% and 42.9%, respectively, under 12.0 dS m^−1^ salinity level in comparison to untreated plants. 

### 2.6. SA and/or SPM Foliar Applications Upregulate the Activity of Antioxidant Enzymes under Saline Conditions

**Figure 6:**
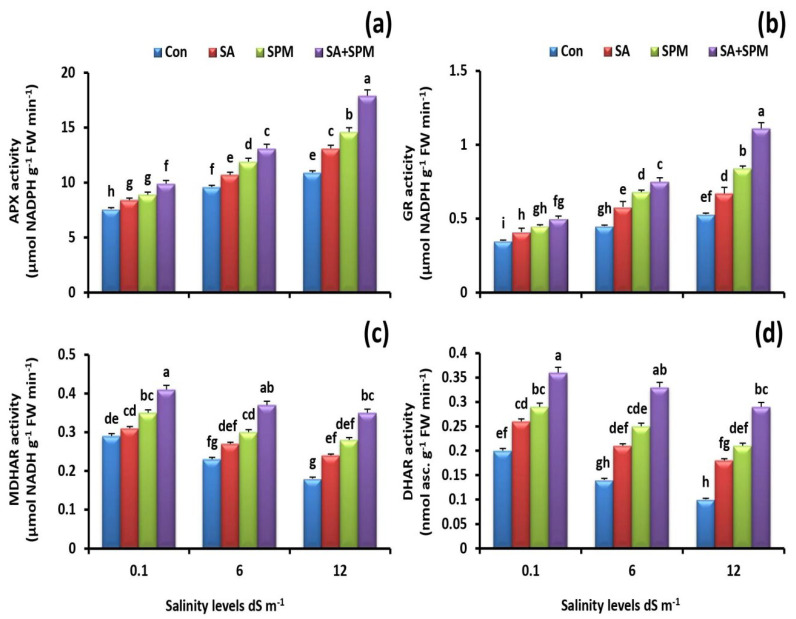
Effect of salicylic acid (SA) and/or spermine (SPM) foliage applications on the activity of (**a**) ascorbate peroxidase (APX), (**b**) glutathione reductase (GR), (**c**) monodehydroascorbate reductase (MDHAR), and (**d**) dehydroascorbate reductase (DHAR) in leaves of wheat plants exposed to non-saline and saline (6.0 and 12.0 dS m^−1^) conditions. The results showed the mean ± SE of four replicates. Different letters indicate significant differences at (*p* < 0.05) level according to Duncan’s test.

To understand how SA and/or SPM ameliorated oxidative damage induced by salinity, the activity of different antioxidant enzymes was assayed. The results showed that while salt treatments improved APX and GR activities, they decreased MDHAR and DHAR activities. However, SA and/or SPM foliage treatments significantly enhanced the activity of APX, GR, MDHAR, and DHAR under saline conditions (Figure 6a–d). In comparison to untreated plants, the combined SA and SPM treatment under 12.0 dS m^−1^ salinity level significantly (*p* < 0.05) increased the activity of APX (64.2%), GR (109.4%), MDHAR (94.4%), and DHAR (190.0%) in the leaves of wheat plants.

### 2.7. Exogenously Applied SA and/or SPM Enhance Antioxidant Molecules Content in Salt-Stressed Wheat Plants

**Figure 7:**
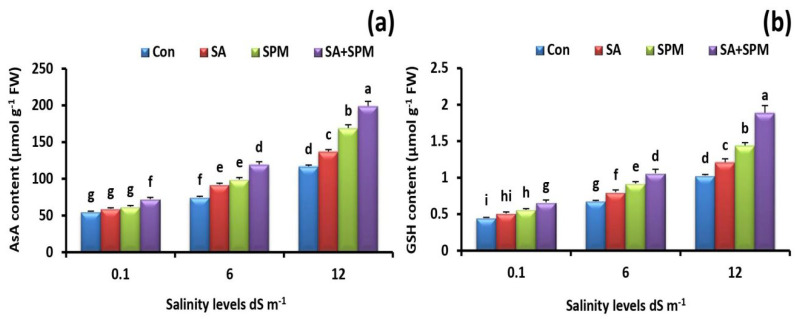
Effect of salicylic acid (SA) and/or spermine (SPM) foliage applications on the content of (**a**) ascorbate (AsA) and (**b**) reduced glutathione (GSH) in leaves of wheat plants exposed to non-saline and saline (6.0 and 12.0 dS m^−1^) conditions. The results showed the mean ± SE of four replicates. Different letters indicate significant differences at (*p* < 0.05) level according to Duncan’s test.

To explain how SA and/or SPM applications eliminate the adverse effects of salt stress, the content of GSH and AsA was quantified. Under salt stress circumstances, a rise in the GSH and AsA contents was seen compared to a non-saline environment. Furthermore, exogenous treatments by SA and/or SPM encouraged their accumulation in plants under salt stress (Figure 7a,b). The best result was obtained when SA and SPM were used together. In comparison to untreated plants, the combined treatment under 12.0 dS m^−1^ salinity level significantly (*p* < 0.05) enhanced the content of GSH by 83.5% and that of AsA by 70.5% in leaves of wheat plants. 

### 2.8. Foliage Applications of SA and/or SPM Improve SA and SPM Concentrations in Salt-Stressed Wheat Plants

**Figure 8:**
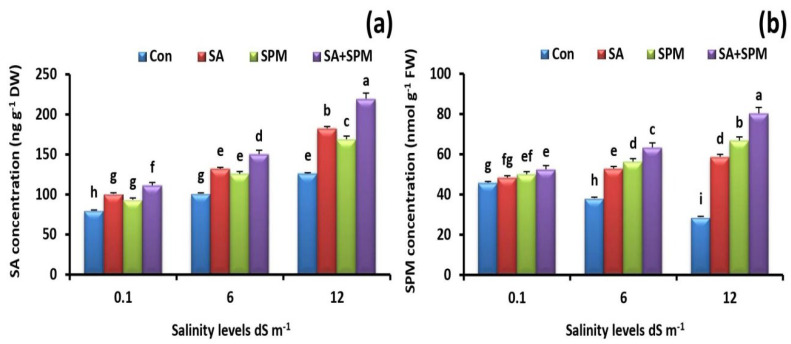
Effect of salicylic acid (SA) and/or spermine (SPM) foliage applications on the endogenous (**a**) salicylic acid and (**b**) spermine concentrations in leaves of wheat plants exposed to non-saline and saline (6.0 and 12.0 dS m^−1^) conditions. The results showed the mean ± SE of four replicates. Different letters indicate significant differences at (*p* < 0.05) level according to Duncan’s test.

Salt stress treatments increased the endogenous concentration of SA in wheat leaves. In addition, SA and/or SPM treatments under saline environments further boosted its level (Figure 8a). Co-application of SA and SPM gave the best response. In comparison to untreated plants, combined treatment under 12.0 dS m^−1^ salinity level significantly (*p* < 0.05) increased the concentration of SA by 73.7% in the leaves of wheat plants. 

Soil salinization markedly reduced the endogenous concentration of SPM, while SA and/or SPM treatments enhanced its level (Figure 8b). When compared to untreated plants, co-application of SA and SPM significantly (*p* < 0.05) improved SPM concentration by 179.8% in the leaves of wheat plants grown under 12.0 dS m^−1^ salinity level. 

## 3. Discussion 

Salt stress is considered a devastating environmental stress that negatively impacts crop productivity [1,23]. It causes osmotic stress, specific ionic impacts, and nutritional imbalances [13,15]. It also disrupts the cell metabolic balance, leading to excessive ROS generation and oxidative stress that severely affects many plant metabolic processes [5,7]. Plant growth regulators such as SA and SPM can act as alleviating-stressor agents and plant master regulators [14,15,19,20], thus they may increase the physiological activity of plants in response to challenging environmental conditions. In this study, we assessed the impact of SA and/or SPM foliar applications on the Calvin cycle enzymes activity, ATP content, root H^+^-pump activity, and antioxidant defense machinery of wheat plants grown under saline conditions. Our results clearly revealed that SA and/or SPM can mitigate salt toxicity by upregulating photosynthetic enzyme activity, enzymatic and non-enzymatic antioxidant systems, and root H^+^-pump activity. This research provides novel insights into the mechanisms of SA- and SPM-mediated amelioration of salt stress on wheat. 

Growth and yield reduction can be used as signs to assess the level of salt-induced injuries in plants [2,24]. In the current study, our results revealed that soil salinization considerably reduced the growth and production of wheat plants which may result from (a) inducing oxidative damage, as indicated by the overproduction and accumulation of H_2_O_2_ and O_2_^•−^, (b) inducing nutritional imbalance that was closely associated with the reduction in N, P, and K^+^ acquisition along with the improvement of Na^+^ accumulation, as well as (c) inactivating the Calvin cycle enzymes. By contrast, in agreement with the previous reports [4,6,7,12,15,18,19,21], we observed that foliage applications of SA and/or SPM significantly ameliorated the negative impacts of soil salinization on wheat growth and production via activating the photosynthetic enzymes, maintaining optimal mineral nutrition through improving roots’ ATP content and H^+^-pump activity, as well as reinforcing antioxidant machinery through suppressing H_2_O_2_ and O_2_^•−^ production, up-regulating antioxidant enzymes (APX, GR, MDHAR, DHAR) activity, and enhancing antioxidant molecules (AsA, GSH) content. These findings clearly prove the effectiveness of SA and/or SPM foliar treatments in attenuating the inhibitory effect of salt stress on plant development by protecting wheat from salt-induced severe ionic and oxidative stresses.

Environmental factors have an impact on the photosynthesis process [5,8,13]. The Calvin cycle is the main pathway by which plants fix carbon [25]. Rubisco, GAPDH, FBPase, and FBA are the key plant enzymes involved in the Calvin cycle. They are important for controlling plant growth and development as well as the abiotic stress response. Rubisco, GAPDH, and FBPase can play important roles in carbon fixation, reduction, and RuBP regeneration processes, respectively [25]. Increased FBA activity can help the Calvin cycle’s assimilation of CO_2_ in plant leaf tissues [26]. The results obtained in this investigation showed that the Calvin cycle enzymes (Rubisco, FBPase, GAPDH, and FBA) were considerably inactivated by salt-stress treatments, whereas SA and/or SPM applications encouraged a rise in their activities under both non-saline and saline circumstances. This behavior may be explained by adaptation to adversity, suggesting that SA and/or SPM treatments may be able to reduce the inhibitory effect of salt stress on plant growth and production by enhancing photosynthetic activity through Calvin cycle regulation. It has been reported that exogenous spermidine alleviates the negative effects of salt stress and increases the activities of ribulose 1,5-bisphosphate carboxylase/oxygenase (Rubisco) and aldolase by upregulating the transcriptions of genes encoding phosphoribokinase and Rubisco [27]. Furthermore, SA improves the efficiency of photosynthetic carbon fixation by activating the photosynthetic enzymes, which restores the supply of CO_2_ to the Rubisco enzyme and helps in overcoming stomatal limitations under stressful conditions [28]. Hence, SPM and SA can exert a positive effect on photosynthesis under saline environments by enhancing the activity of Calvin cycle enzymes. 

In the current study, it was displayed that saline conditions induced alterations in ion homeostasis as shown by higher Na^+^ accumulation and lower N, P, and K^+^ acquisition, moreover, this effect was most marked at high salinity levels. On the contrary, SA and/or SPM foliar treatments relieved the adverse effects caused by salt stress and significantly improved nutrient acquisition (N, P, K^+^) in wheat grains, indicating their regulatory role in enhancing mineral nutrition uptake, accumulation, and translocation. Furthermore, exogenous SA and/or SPM applications ameliorated the deleterious injuries of salinity and reduced the Na^+^ accumulation that might be linked with their roles in H_2_O_2_ and O_2_^•−^ elimination. Our results are in line with previous findings reported by other researchers who have stated that SPM can decrease K^+^ excretion and Na^+^ uptake in salt-stressed plants [18,21,29]. Furthermore, SA’s beneficial effects on maintaining ionic homeostasis may be directly related to its capacity to prevent salt-induced K^+^ leakage via depolarization-activated-outward-rectifying K^+^ channels [30,31]. The results obtained in this trial also revealed that foliage applications of SA and/or SPM resulted in improved ATP content and H^+^-pump activity in the roots of salt-stressed wheat plants. These results may suggest that the higher H^+^-ATPase activity could be connected to SA and SPM’s protective effects on nutrient uptake. Our suggestion is consistent with previous reports, which demonstrated that the H^+^-ATPase activity is directly correlated with the mineral status in stressed plants [15,32]. Based on our research, SA and/or SPM may be able to ameliorate the growth and yield reduction under saline environments by modifying the ionic status of the plant.

Under salt stress conditions, plants usually accumulate large amounts of ROS, which lead to oxidative damage [5]. In the current work, we found that wheat leaves accumulated more ROS (H_2_O_2_ and O_2_^•−^) by increasing salinity level, whereas SA and/or SPM applications help plants tolerate oxidative stress by improving H_2_O_2_ and O_2_^•−^ removal. Strong evidence has demonstrated that the tetra-amine SPM can successfully scavenge ROS due to its polycationic structure, making it a potent free radical scavenger [18,19,20]. Similarly, SA may be taken into account because it may act directly as an antioxidant, scavenging ROS and/or indirectly modulating redox balance through the activation of antioxidant responses to ameliorate oxidative stress-induced damage in abiotic stresses [33]. Previous studies have also shown that the reduction in ROS via SA treatment may be related to the fact that SA is a direct scavenger of ROS produced during stressful conditions, as well as stimulating the enzymatic and non-enzymatic antioxidant defense components [7,34]. Hence, exogenously applied SA and/or SPM may effectively avoid oxidative damage by deactivating and scavenging harmful free radicals. 

To deal with oxidative damage and lessen excessive ROS formation, plants have evolved defense mechanisms that include antioxidants with enzymatic or non-enzymatic activity [5,35]. In the current study, a change in the activity of antioxidant enzymes was found in wheat plants exposed to oxidative damage brought by salt stress. The reason behind that might be due to the increased ROS-mediated oxidative stress and cell injury under saline environments in wheat plants. Moreover, this antioxidant strategy was altered by SA and/or SPM treatments in salt-stressed plants. Our results may suggest that salt stress induced oxidative damage has been controlled by up-regulating APX, GR, MDHAR, and DHAR activities. As a H_2_O_2_ scavenger, APX converts H_2_O_2_ into H_2_O and upregulates the antioxidant defense mechanism at the cellular level upon exposure to salt stress. Along with APX activity, DHAR, MDHAR and GR are the important ROS scavenger [5,35]. Interestingly, this increment in the APX, GR, MDHAR, and DHAR activities of salt-stressed treated plants could be an adaptive way to overcome salt damage by reducing toxic levels of ROS and providing protection against oxidative stress. A positive correlation between SA application and antioxidant enzyme activity under stressful conditions has also been reported by [7,36,37]. Previous studies have demonstrated that SA-induced changes in the antioxidant enzyme activity can be linked to H_2_O_2_ detoxification [12,38]. Regarding SPM application, it was reported that SPM can act as a signaling molecule, scavenge free radicals, and positively regulate the activity of antioxidant enzymes under saline environments [19,20]. Some researchers have proved exogenous SPM can increase the transcription level of antioxidant enzymes and then provoke antioxidant enzyme activity in plants under stressful conditions [18,39]. Overall, SA and SPM may have a role in improving wheat salt tolerance due to their ability to prevent ROS bursts. 

In addition to enhancing the activity of antioxidant enzymes, SA and/or SPM treatments also contribute to AsA–GSH cycle modulation. The AsA-GSH cycle is an important antioxidant system in plants. AsA is a small molecule antioxidant, which in vivo can react directly with ROS to eliminate it. AsA also plays an important role in ROS scavenging as a substrate for enzymes. In addition to the direct scavenging of free radicals, GSH also plays an important role in regenerating antioxidant substances, such as AsA [40,41]. In the present study, our results revealed that SA and/or SPM applications to stressed plants maintained AsA and GSH regeneration, which could be due to the increment in APX, MDHAR, DHAR, and GR activities. These results may suggest that the ability of treated plants to grow and survive in a saline environment is largely controlled by their ability to use antioxidant machinery. This suggestion is consistent with that of You and Chan [35], who hypothesized that the AsA-GSH cycle; which includes APX, MDHAR, DHAR, and GR as well as AsA and GSH as enzymatic and non-enzymatic components, is necessary for H_2_O_2_ elimination. Evidence showed the role of SA in the alleviation of salt stress in plants by boosting the activity of AsA-GSH cycle-related enzymes as well as the content of AsA and GSH [42]. Indeed, SA’s beneficial effects on maintaining AsA and GSH pool in salt-stressed plants may be directly related to its capacity to catalyze the formation of AsA and GSH or upregulate the activity of enzymes linked to the AsA-GSH cycle [7,43,44]. Furthermore, the use of SPM can also affect the production of AsA and GSH. In this respect, Ahangera et al. [19] revealed that the up-regulation of the enzymatic components of the AsA-GSH cycle due to foliar SPM application assisted the stressed plants to neutralize excess H_2_O_2_ and maintain the redox homeostasis by generating GSH and AsA. Our results are also supported by other studies where SPM reduces salt injuries by improving the content of GSH and the activity of GR, APX, and MDHAR as well as reducing ROS production [21,45]. Taken together, these findings imply that SA and/or SPM treatments resulted in a unique antioxidant profile. Antioxidant defense machinery, including antioxidant enzymes and antioxidant compounds, is tightly coordinated to keep the cellular redox balance at its ideal level and stimulate ROS detoxification. In conclusion, this improvement in antioxidant machinery could maintain plant physiological and biochemical processes, resulting in better plant growth and development, and tolerance to salt stress. This is consistent with previous reports, which demonstrated that SA and/or SPM capability in reducing ROS (H_2_O_2_ and O_2_^•−^) accumulation enhanced the photosynthetic efficiency, mineral uptake, plant growth, and biomass production under stressful conditions [6,12,15,18,19,45]. 

In addition to improving plant growth and development, phytohormones such as SA and SPM can also enhance plant stress tolerance [18,19,20,21,39,46,47]. In the current investigation, we displayed that endogenous SA and SPM concentrations in wheat leaves were significantly increased by SA and/or SPM foliage treatments under saline conditions, indicating their potential role as stress-relieving agents. In line with our findings, previous research by Canales [48] demonstrated that SA regulated PA biosynthesis through changes in PA gene expression. SA treatment decreased the level of putrescine (*Put*) under stressful conditions while increasing those of SPM. Moreover, strong evidence has revealed that the exogenous application of SPM stimulates the stress-responsive genes of endogenous phytohormones, especially SA, as confirmed by the pronounced transcript levels of SA-related genes (OsPR1, OsPR2 and OsNPR1) [49]. Most probably, the key mechanism is that the exogenous supply of SA or SPM stimulates the endogenous SA and SPM levels, which may help plants boost their tolerance to outside environmental stressors, improving the morpho-physiological and biochemical attributes of wheat [7,15,50]. 

**Figure 9:**
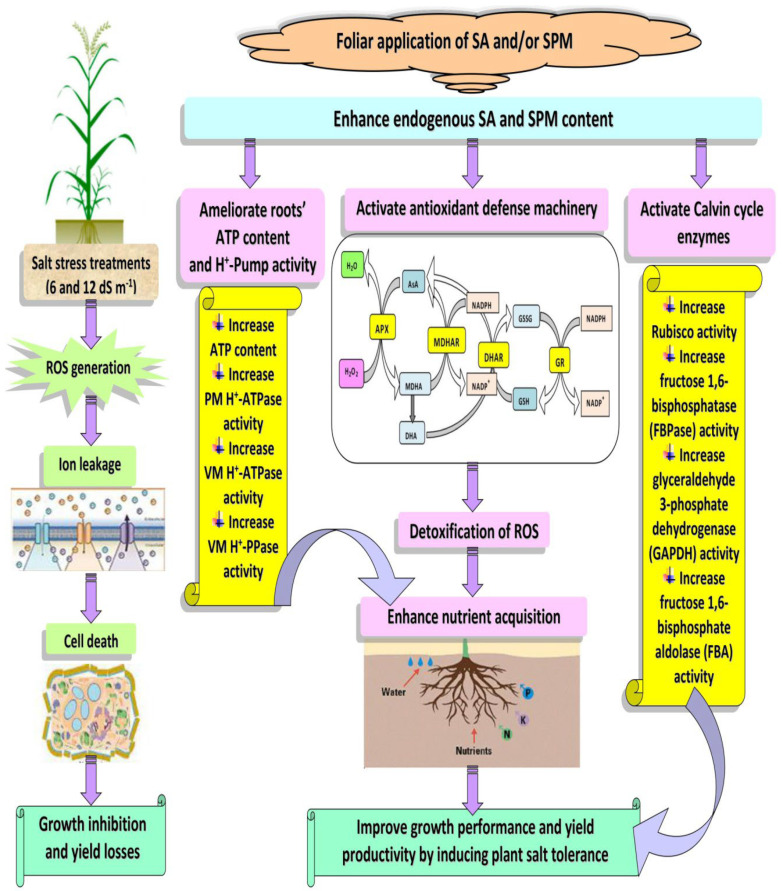
A model showing salt stress induces oxidative stress in wheat plants by increasing reactive oxygen species (ROS) generation. Meanwhile, salicylic acid (SA) and/or spermine (SPM) foliage applications reduce salt stress damage to the plants and improve growth performance and yield productivity by increasing endogenous SA and SPM concentrations that activate Calvin cycle enzymes and antioxidant defense machinery as well as ameliorate roots’ ATP content and H^+^-pump activity.

Based on our findings, we can suggest that SA and/or SPM foliar applications increase endogenous SA and SPM levels that alleviate salt-stress symptoms in wheat plants by enhancing Calvin cycle enzymes activity, reinforcing antioxidant machinery through suppressing ROS production, as well as maintaining optimal mineral nutrition through improving roots’ ATP content and H^+^-pump activity (Figure 9). Indeed, this study provides specific insight and new ideas for the effectiveness of SA and SPM coupling treatment in mediating salt tolerance. At the same time, it is important for basic and applied plant physiology and agricultural production applications.

## 4. Materials and Methods

### 4.1. Plant Material and Experimental Design

A pot experiment was conducted at the greenhouse of the Plant Physiology Department, Faculty of Agriculture, Cairo University, Egypt. The experiment was repeated twice, on September 10 of 2020 and 2021. Wheat (*Triticum aestivum* L. cv. Shandawel 1) grains were kindly supplied by the Wheat Research Department, Agriculture Research Center, Egyptian Ministry of Agriculture. The pots were 30 cm in diameter and 35 cm in height and contained 15 kg of clay loamy soil (sand 37%, silt 28%, clay 35%). NPK fertilizations were carried out according to the Ministry of Agriculture recommendations. Table 1 shows the soil chemical analysis, which was performed according to the procedures of Cottenie et al. [51]. Pots were divided into three groups before sowing. The first group was assigned as control (non-saline; 0.1 dS m^−1^) and the other two groups as two levels of salinity treatment (6.0 and 12.0 dS m^−1^ salinity level; obtained by adding to the soil a mixture of NaCl, CaCl_2_, and MgSO_4_ at the molar ratio of 2:2:1, respectively).

The wheat plants at 50 days old (vegetative stage) and 100 days old (grain filling stage) from each salinity level were foliar sprayed with 0.00 (distilled water; DW), 100 mgL^−1^ SA, 30 mgL^−1^ SPM, and 100 mgL^−1^ SA + 30 mgL^−1^ SPM. The concentrations of SA and SPM were chosen based on the results of a preliminary experiment. Tween-20 (0.05%) was used as a surfactant at the time of treatment.

The experimental layout was completely randomized design with two factors: three levels of salinity [0.1 dS m^−1^ (non-saline), 6.0 and 12.0 dS m^−1^], and four spraying treatments [0.00 (distilled water; DW), 100 mgL^−1^ SA, 30 mgL^−1^ SPM, and 100 mgL^−1^ SA + 30 mgL^−1^ SPM]. Each treatment was replicated four times. The plants were sampled after 75 days of sowing to assess the total leaf area (using a portable leaf area meter (LI-COR 3000, Lambda Instruments Corporation, Lincoln, NE, USA), and shoot dry weight. After maturation, the number of grains and grain yield were estimated. Data were collected from four replicates, and each replicate includes six plants gathered from the same pot.

The following physiological and biochemical traits were determined in 75-day-old (after 25 days of SA and/or SPM first applications) wheat leaves. Data were collected from four replicates, each of which contained six plants gathered from the same pot.

### 4.2. Assay of Calvin Cycle Enzymes

Calvin cycle enzymes [ribulose diphosphate carboxylase/oxygenase (Rubisco), fructose 1,6-bisphosphatase (FBPase), glyceraldehyde 3-phosphate dehydrogenase (GAPDH), and fructose 1,6-bisphosphate aldolase (FBA)] activity was assessed by ELISA kits (Yaji Biotech, Shanghai, China). In an extraction buffer containing 0.05 mM Tris-HCl and 0.1 M phosphate buffer; pH 7.4, wheat leaf samples were ground. They were then under centrifugation procedure (3000× *g* for 15 min at 4 °C). The enzyme activity assay was conducted using the supernatant. The test sample (the standard and the horseradish peroxidase-conjugate reagent) was placed in the microplate wells. The Rubisco antibody was already present in the microplate wells for determining Rubisco activity. After incubating for 60 min at 37 °C, the samples were washed. Peroxidase transformed the substrate 3,3′,5,5′- tetramethylbenzidine to blue, and acid action converted it to yellow. At 450 nm, the color’s intensity was determined. The optical density of the samples was then compared to the standard curve to assess Rubisco activity. Other enzymes’ activities were also measured in the same way. Enzyme activity (U) is defined as the quantity of enzyme required to convert 1 µmol of the substrate in 1 min under optimal conditions.

### 4.3. Determination of Mineral Element Concentrations 

Dried grains (0.5 g) were ground and digested in a solution of boiling perchloric acid and hydrogen peroxide for 8 h, resulting in a transparent solution. Nitrogen concentration was determined using the modified micro-Kjeldahl method as described by Pregl [52]. The vanadomolybdophosphoric method was used to determine phosphorus concentration as performed by Kacar and Inal [53]. A flame photometer (ELE UK) was used to measure the potassium and sodium concentrations. An atomic-absorption spectrophotometer (Unicam 989-AA Spectrometer-UK) was used to determine the iron and copper concentrations.

### 4.4. ATP Content Determination 

ATP was extracted as previously explained by Stewart and Guinn [54]. According to the manufacturer’s instructions, ATP content was measured using an ATP Colorimetric/Fluorometric Assay Kit (BioVision, Milpitas, CA, USA).

### 4.5. Plasma Membrane (PM) and Vacuole Membrane (VM) Separation as Well as Measurement of H^+^-Pump Activity

The wheat roots were cut 2 cm from the tip and then rinsed with deionized water. The plasma and vacuole membranes were isolated using the method described by Yan et al. [55]. In a cold grinding medium containing (Hepes-Tris 60 mM, pH 7.5, source 300 mM, EDTA 5 mM, EGTA 0.5 mM, DTT 2 mM, 1.5% PVP, PMSF 2 mM, DTT 2 mM, BSA 0.1%), the excised roots (10 g) were thoroughly chopped and homogenized (1/3, *w/v*). The homogenate was centrifuged at 13,000× *g* for 20 min after being filtered through four layers of cheesecloth. The supernatant was centrifuged at 80,000× *g* for 30 min in a discontinuous sucrose gradient (containing 45%, 33%, and 15% (*m/v*) sucrose solution). Five mL of centrifuged sediment was collected at the interface between 15 and 33% and 33 and 45% gradients, respectively. The gradient centrifugation buffer (HEPES tris 20 mM, pH 7.5, EDTA 5 mM, EGTA 0.5 mM) was used to dilute the 15–33% gradient to twice the volume, while the gradient centrifugation buffer was used to dilute the 33–45% gradient to four times the volume. The supernatant was removed after 100,000× *g* of centrifugation for 1 h. To obtain the VM and PM microcapsules, the precipitates were suspended in 0.5 mL of suspension (HEPES tris 20 mM, pH 7.5, sucrose 300 mM, EGTA 0.5 mM, MgCl_2_⋅6H_2_O 0.5 mM). Protein concentration was determined using the Coomassie brilliant blue method and BSA as a standard. 

The activities of H^+^-ATPase and H^+^-PPase were determined using the method described by Wang and Sze [56]. In brief, 15–20 μL tonoplast vesicles were added to 400 μL of the reaction medium, which contained 30 mM Hepes–Tris (pH 6.0, pH 8.5 for H^+^-PPase assay), 3.0 mM MgSO_4_, 0.5 mM NaN_3_, 0.1 mM Na_3_VO_4_, 50 mM KCl, 0.1 mM ammonium molybdate, and 3.0 mM ATP (or 2.0 mM Na_4_PPi for H^+^-PPase assay). After 20 min at 37 °C, 50 μL of TCA was added to stop the reaction. The method of Ohnishi et al. [57] was used to calculate the amount of inorganic phosphate released from the hydrolysis of ATP or PP.

### 4.6. Determination of Hydrogen Peroxide (H_2_O_2_) and Superoxide Radical (O_2_^•−^) Content 

To estimate H_2_O_2_ and O_2_^•−^, 0.1 g fresh wheat leaves were homogenized in 900 µL buffer according to the instructions provided in the H_2_O_2_ and O_2_^•−^ kits, using the methods described by Nawaz et al. [58] and Gao et al. [59], respectively. The contents of H_2_O_2_ and O_2_^•−^ were measured at wavelengths of 405 and 550 nm, respectively.

### 4.7. Estimation of Antioxidant-Defense Enzymes Activity as Well as Reduced (GSH) Glutathione and Reduced (AsA) Ascorbate Content 

Fresh wheat leaves (0.5 g) were homogenized in 5 mL of ice-cold 100 mM phosphate buffer (pH 7.4) containing 1% polyvinyl pyrrolidine and 1 mM EDTA and then centrifuged at 15,000× *g* for 10 min at 25 °C. For the assays, the supernatant was gathered and used. According to the method of Ramel et al. [60], the APX activity was determined by monitoring the decrease in absorbance at 290 nm caused by ascorbate oxidation. The MDHAR activity was determined by observing NADH oxidation at 340 nm [61]. The DHAR activity was measured by examining ascorbate formation at 265 nm [62]. The GR activity was assayed by detecting NADPH oxidation at 340 nm [63]. The amount of AsA and GSH was determined according to Hernandez et al. [64].

### 4.8. Measuring the Endogenous SA and SPM Contents 

*Wheat leaves were washed thoroughly* and very carefully to remove any SA and SPM residues, before being used. SA’s concentration was measured in accordance with Enyedi et al. [65] and Seskar et al. [66]. Frozen dried leaf samples (0.3 g) were ground with liquid nitrogen before being extracted with methanol (90 and 100%) by centrifugation at 12,000× *g* for 15 min at 4 °C. The dried residue was dissolved in 5% trichloroacetic acid and centrifuged at 10,000× *g* for 10 min. The supernatant was partitioned with 49.5:49.5:1 *v/v* ethyl acetate/cyclopentane/isopropanol. For high-performance liquid chromatography (HPLC), the organic phase was collected and dried in nitrogen. The powder was resuspended in methanol, and the homogenate was filtered through a needle filter into a sample bottle. A standard SA sample (Solarbio, Beijing, China) was used; chromatography was performed on a reverse-phase HPLC column (ABZ1, 250.0 × 34.6 mm; Supelco, Buchs, Switzerland). 

In addition, wheat fresh leaf samples (0.3 g) were used for SPM quantification. The extraction, benzoylation and HPLC (Waters, Milford, MA, USA) analyses were performed according to Naka et al. [67]. In brief, after grinding 0.3 g of fresh leaf samples, 1.5 mL of 5% (*v/v*) cold perchloric acid was applied, then the mixture was placed in plastic tubes and kept on ice for 1 h. The supernatants were mixed and filtered using a filter syringe (pore size, 0.2 μm) after being centrifuged at 15,000× *g* for 30 min at 4 °C. One mL of 2 N NaOH was added to 1.5 mL of plant extract, mix by vortex, and then 10 μL of benzoyl chloride was added, mixed, and incubated at room temperature for 20 min. Saturated sodium chloride in a volume of 2 mL was added. Then, 2 mL of diethyl ester was added and the phases were separated using a 3000× *g* centrifuge for 10 min at 4 °C. The diethyl ether phase (1.5 mL) was evaporated, and the residue was then re-dissolved in 50 μL of methanol. The benzoylated samples were analyzed with HPLC at a flow rate of 1 mL/min using a reverse-phase column (4.6 × 250 mm, TSK-GEL ODS-80Ts, Tosoh, Tokyo, Japan) and detected at 254 nm. One cycle of the run resume consisted of 60 min at a flow rate of 1 mL/min at 30 °C; i.e., 42% acetonitrile for 25 min for PA separation, increased up to 100% acetonitrile during 3 min, 100% acetonitrile for 20 min for washing, decreased down to 42% acetonitrile during 3 min, then 42% acetonitrile for 9 min.

### 4.9. Statistical Data Analysis 

A completely randomized design with four replicates per treatment was used. Because the results of the two growing seasons followed a similar trend, a combined analysis was performed. All measured parameters were statistically analyzed by the two-way ANOVA test, where the first factor was the salt treatments, and the second was the foliar application treatments. Differences between the treatments were tested by Duncan’s test at a level of significance *p* < 0.05. The data are presented as means ± standard error (SE). The SAS software (SAS Inc., Cary, NC, USA) was used for the statistical analysis.

## 5. Conclusions

Our results reveal that promoting an antioxidant defense system, motivating plant nutrient acquisition, and activating Calvin cycle enzymes in stressed SA- and SPM-treated plants could improve wheat growth and productivity. Indeed, SA and/or SPM foliar applications increased endogenous SA and SPM levels, which ameliorated the negative impact of saline environments on wheat growth and development via activating the Calvin cycle enzymes [ribulose diphosphate carboxylase/oxygenase (Rubisco), fructose 1,6-bisphosphatase (FBPase), glyceraldehyde 3-phosphate dehydrogenase (GAPDH), and fructose 1,6-bisphosphate aldolase (FBA)] activity, maintaining optimal mineral nutrition through improving roots’ ATP content and H^+^-pump activity, as well as reinforcing antioxidant machinery through suppressing H_2_O_2_ and O_2_^•−^ production, up-regulating antioxidant enzymes (APX, GR, MDHAR, DHAR) activity, and enhancing antioxidant molecules (AsA, GSH) content. Hence, phytohormones such as SA and SPM can play key roles in enhancing wheat salt tolerance. The best result was obtained when SA and SPM were used together. It is praiseworthy that the combined treatment of SA and SPM as an environmentally friendly approach could be used as a novel tool against harsh environmental conditions in agronomic and horticultural crops.

## Figures and Tables

**Table 1:** Basic characteristics of the soil used in this experiment.

Salinity Levels EC (dS m^−1^)	pH	HCO_3_^−^ + CO_3_^2−^ (mg kg^−1^)	Cl^−^ (mg kg^−1^)	SO_4_^2−^ (mg kg^−1^)	Ca^2+^ (mg kg^−1^)	Mg^2+^ (mg kg^−1^)	Na^+^ (mg kg^−1^)	K^+^ (mg kg^−1^)
0.1	7.2	213.5	324.0	430.7	92.2	41.4	3.7	31.4
6	7.5	263.6	1173.4	996.9	398.5	173.9	306.7	39.7
12	7.8	275.4	1987.8	1686.1	886.5	314.5	808.6	52.6

## Data Availability

The data presented in this study are available in article.

## References

[1] Zörb C., Geilffus C.M., Dietz K.J. (2019). Salinity and crop yield. Plant Biol..

[2] Talaat N.B., Shawky B.T. (2012). Influence of arbuscular mycorrhizae on root colonization, growth and productivity of two wheat cultivars under salt stress. Arch. Agron. Soil Sci..

[3] Amirbakhtiar N., Ismaili A., Ghaffari M.-R., Mirdar Mansuri R., Sanjari S., Shobbar Z.-S. (2021). Transcriptome analysis of bread wheat leaves in response to salt stress. PLoS ONE.

[4] Es-sbihi F.Z., Hazzoumi Z., Aasfar A., Joutei K.A. (2021). Improving salinity tolerance in *Salvia officinalis* L. by foliar application of salicylic acid. Chem. Biol. Technol. Agric..

[5] Talaat N.B., Mostafa A.A., El-Rahman S.N.A. (2022). A novel plant growth-promoting agent mitigates salt toxicity in barley (*Hordeum vulgare* L.) by activating photosynthetic, antioxidant defense, and methylglyoxal detoxification machineries. J. Soil Sci. Plant Nutr..

[6] Shamili M., Ghalati R.E., Samari F. (2021). The impact of foliar salicylic acid in salt-exposed guava (*Psidium Guajava* L.) seedlings. Int. J.Fruit Sci..

[7] Talaat N.B., Todorova D. (2022). Antioxidant machinery and glyoxalase system regulation confers salt stress tolerance to wheat (*Triticum aestivum* L.) plants treated with melatonin and salicylic Acid. J. Soil Sci. Plant Nutr..

[8] Shah F.A., Ni J., Tang C., Chen X., Kan W., Wu L. (2021). Karrikinolide alleviates salt stress in wheat by regulating the redox and K^+^/Na^+^ homeostasis. Plant Physiol. Biochem..

[9] Wang F., Wang C., Sun Y., Wang N., Li X., Dong Y., Yao N., Liu X., Chen H., Chen X. (2016). Overexpression of vacuolar proton pump ATPase (V-H^+^-ATPase) subunits B, C and H confers tolerance to salt and saline-alkali stresses in transgenic alfalfa (*Medicago sativa* L.). J. Integr. Agric..

[10] Silva P., Façanha A.R., Tavares R.M., Gerós H. (2019). Role of tonoplast proton pumps and Na^+^/H^+^ antiport system in salt tolerance of *Populus euphratica* Oliv. J. Plant Growth Regul..

[11] Queirós F., Fontes N., Silva P., Almeida D., Maeshima M., Gerós H., Fidalgo F. (2009). Activity of tonoplast proton pumps and Na^+^/H^+^ exchange in potato cell cultures is modulated by salt. J. Exp. Bot..

[12] Bukhat S., Manzoor H., Athar H., Zafar Z.U., Azeem F., Rasul S. (2020). Salicylic acid induced photosynthetic adaptability of *Raphanus sativus* to salt stress is associated with antioxidant capacity. J. Plant Growth Regul..

[13] Hoang H.L., de Guzman C.C., Cadiz N.M., Hoang T.T.H., Tran D.H., Rehman H. (2020). Salicylic acid and calcium signaling induce physiological and phytochemical changes to improve salinity tolerance in red amaranth (*Amaranthus tricolor* L.). J. Soil Sci. Plant Nutr..

[14] Tanjimul Islam A.T.M., Ullah H., Himanshu S.K., Tisarum R., Cha-um S., Datta A. (2022). Effect of salicylic acid seed priming on morpho-physiological responses and yield of baby corn under salt stress. Sci. Hortic..

[15] Talaat N.B., Shawky B.T. (2022). Synergistic effects of salicylic acid and melatonin on modulating ion homeostasis in salt-stressed wheat (*Triticum aestivum* L.) plants by enhancing root H^+^-pump activity. Plants.

[16] Todorova D., Talaat N.B., Katerova Z., Alexieva V., Shawky B.T., Ahmad P. (2016). Polyamines and brassinosteroids in drought stress responses and tolerance in plants. Water Stress and Crop Plants: A Sustainable Approach, Volume 2.

[17] Talaat N.B. (2020). 24-Epibrassinolide and spermine combined treatment sustains maize (*Zea mays* L.) drought-tolerance by improving photosynthetic efficiency and altering phytohormones Profile. J. Soil Sci. Plant Nutr..

[18] Xu J., Yang J., Xu Z., Zhao D., Hu X. (2020). Exogenous spermine-induced expression of *SlSPMS* gene improves salinity–alkalinity stress tolerance by regulating the antioxidant enzyme system and ion homeostasis in tomato. Plant Physiol. Biochem..

[19] Ahangera M.A., Qin C., Maodong Q., Dong X.X., Ahmad P., Abd Allah E.F., Zhang L. (2019). Spermine application alleviates salinity induced growth and photosynthetic inhibition in *Solanum lycopersicum* by modulating osmolyte and secondary metabolite accumulation and differentially regulating antioxidant metabolism. Plant Physiol. Biochem..

[20] Islam M.A., Jin-huan P., Fan-wei M., Ya-wen L., Ning X., Chao Y., Jun L. (2020). Putrescine, spermidine, and spermine play distinct roles in rice salt tolerance. J. Integr. Agric..

[21] Geng W., Qiu Y., Peng Y., Zhang Y., Li Z. (2021). Water and oxidative homeostasis, Na^+^/K^+^ transport, and stress-defensive proteins associated with spermine-induced salt tolerance in creeping bentgrass. Environ. Exp. Bot..

[22] Hasan M.M., Skalicky M., Jahan M.S., Hossain M.N., Anwar Z., Nie Z.F., Alabdallah N.M., Brestic M., Hejnak V., Fang X.W. (2021). Spermine: Its emerging role in regulating drought stress responses in plants. Cells.

[23] Royo A., Abió D. (2003). Salt tolerance in durum wheat cultivars. Span. J. Agric. Res..

[24] Acosta-Motos J.R., Ortuno M.F., Bernal-Vicente A., Diaz-Vivancos P., Sanchez-Blanco M.J., Hernandez J.A. (2017). Plant responses to salt stress: Adaptive mechanisms. Agronomy.

[25] Sharma S., Joshi J., Kataria S., Verma S.K., Chatterjee S., Jain M., Pathak K., Rastogi A., Brestic M. (2020). Regulation of the Calvin cycle under abiotic stresses: An overview. Plant Life under Changing Environment.

[26] Lv G.Y., Guo X.G., Xie L.P., Xie C.G., Zhang X.H., Yang Y., Xiao L., Tang Y.Y., Pan X.L., Guo A.G. (2017). Molecular characterization, gene evolution, and expression analysis of the fructose-1, 6-bisphosphate aldolase (FBA) gene family in wheat (*Triticum aestivum* L.). Front. Plant Sci..

[27] El Sayed A.I., El-hamahmy M.A.M., Rafudeen M.S., Ebrahim M.K.H. (2019). Exogenous spermidine enhances expression of Calvin cycle genes and photosynthetic efficiency in sweet sorghum seedlings under salt stress. Biol. Plant..

[28] Shao R.X., Xin L.F., Guo J.M., Zheng H.F., Mao J., Han X.P., Jia L., Jia S.J., Du C.G., Song R. (2018). Salicylic acid-induced photosynthetic adaptability of *Zea mays* L. to polyethylene glycol-simulated water deficit is associated with nitric oxide signaling. Photosynthetica.

[29] Shabala S., Cuin T.A., Pottosin I.I. (2007). Polyamines prevent NaCl induced K^+^ efflux from pea mesophyll by blocking non-selective cation channels. FEBS Lett..

[30] Gharbi E., Lutts S., Dailly H., Quinet M. (2018). Comparison between the impacts of two different modes of salicylic acid application on tomato (*Solanum lycopersicum*) responses to salinity. Plant Signal. Behav..

[31] Hongna C., Leyuan T., Junmei S., Xiaori H., Xianguo C. (2021). Exogenous salicylic acid signal reveals an osmotic regulatory role in priming the seed germination of *Leymus chinensis* under salt-alkali stress. Environ. Exp. Bot..

[32] Inada M., Ueda A., Shi W., Takabe T.A. (2005). stress-inducible plasma membrane protein 3 (AcPMP3) in a monocotyledonous halophyte, *Aneurolepidium chinense*, regulates cellular Na^+^ and K^+^ accumulation under salt stress. Planta.

[33] Hediji H., Kharbech O., Ben Massoud M., Boukari N., Debez A., Chaibi W., Chaoui A., Djebali W. (2021). Salicylic acid mitigates cadmium toxicity in bean (*Phaseolus vulgaris* L.) seedlings by modulating cellular redox status. Environ. Exp. Bot..

[34] Colak N., Kurt-Celebi A., Fauzan R., Torun H., Ayaz F.A. (2021). The protective effect of exogenous salicylic and gallic acids ameliorates the adverse effects of ionizing radiation stress in wheat seedlings by modulating the antioxidant defence system. Plant Physiol. Biochem..

[35] You J., Chan Z. (2015). ROS regulation during abiotic stress responses in crop plants. Front. Plant Sci..

[36] Hussain S.J., Khan N.A., Anjum N.A., Masood A., Khan M.I.R. (2021). Mechanistic elucidation of salicylic acid and sulphur-induced defence systems, nitrogen metabolism, photosynthetic, and growth potential of mungbean (*Vigna radiata*) under salt stress. J. Plant Growth Regul..

[37] Hasanuzzaman M., Ahmed N., Saha T., Rahman M., Rahman K., Alam M.M., Rohman M.M., Nahar K. (2022). Exogenous salicylic acid and kinetin modulate reactive oxygen species metabolism and glyoxalase system to confer waterlogging stress tolerance in soybean (*Glycine max* L.). Plant Stress.

[38] Nigam B., Dubey R.S., Rathore D. (2022). Protective role of exogenously supplied salicylic acid and PGPB (*Stenotrophomonas* sp.) on spinach and soybean cultivars grown under salt stress. Sci. Hortic..

[39] Talaat N.B., Ibrahim A.S., Shawky B.T. (2022). Enhancement of the expression of *ZmBZR1* and *ZmBES1* regulatory genes and antioxidant defense genes triggers water stress mitigation in maize (*Zea mays* L.) plants treated with 24-epibrassinolide in combination with spermine. Agronomy.

[40] Fan Y., Bose J., Zhou M., Shabala S., Shabir H.W., Mohammad A.H. (2016). ROS production, scavenging, and signaling under salinity stress. Managing Salt Tolerance in Plants: Molecular and Genomic Perspectives.

[41] Yao M., Ge W., Zhou Q., Zhou X., Luo M., Zhao Y., Wei B., Ji S. (2021). Exogenous glutathione alleviates chilling injury in postharvest bell pepper by modulating the ascorbate-glutathione (AsA-GSH) cycle. Food Chem..

[42] Kaya C., Ashraf M., Alyemeni M.N., Ahmad P. (2020). The role of endogenous nitric oxide in salicylic acid-induced up-regulation of ascorbate-glutathione cycle involved in salinity tolerance of pepper (*Capsicum annuum* L.) plants. Plant Physiol. Biochem..

[43] Kamran M., Xie K., Sun J., Wang D., Shi C., Lu Y., Gu W., Xu P. (2020). Modulation of growth performance and coordinated induction of ascorbate-glutathione and methylglyoxal detoxification systems by salicylic acid mitigates salt toxicity in choysum (*Brassica parachinensis* L.). Ecotoxicol. Environ. Saf..

[44] Zaid A., Mohammad F., Wani S.H., Siddique K.M.H. (2019). Salicylic acid enhances nickel stress tolerance by up-regulating antioxidant defense and glyoxalase systems in mustard plants. Ecotoxicol. Environ. Saf..

[45] Shah A.A., Riaz L., Siddiqui M.H., Nazar R., Ahmed S., Yasin N.A., Ali A., Mukherjee S., Hussaan M., Javad S. (2022). Spermine-mediated polyamine metabolism enhances arsenic-stress tolerance in *Phaseolus vulgaris* by expression of zinc-finger proteins related genes and modulation of mineral nutrient homeostasis and antioxidative system. Environ. Pollut..

[46] Yousefvand P., Sohrabi Y., Heidari G., Weisany W., Mastinu A. (2022). Salicylic acid stimulates defense systems in allium hirtifolium grown under water deficit stress. Molecules.

[47] Biareh V., Shekari F., Sayfzadeh S., Zakerin H., Hadidi E., Beltrão J.G.T., Mastinu A. (2022). Physiological and qualitative response of *Cucurbita pepo* L. to salicylic acid under controlled water stress conditions. Horticulturae.

[48] Canales F.J., Montilla-Bascón G., Rispail N., Prats E. (2019). Salicylic acid regulates polyamine biosynthesis during drought responses in oat. Plant Signal. Behav..

[49] Basit F., Bhat J.A., Ulhassan Z., Noman M., Zhao B., Zhou W., Kaushik P., Ahmad A., Ahmad P., Guan Y. (2022). Seed priming with spermine mitigates chromium stress in rice by modifying the ion homeostasis, cellular ultrastructure and phytohormones Balance. Antioxidants.

[50] Igarashi K., Kashiwagi K. (2019). The functional role of polyamines in eukaryotic cells. Int. J. Biochem. Cell Biol..

[51] Cottenie A., Verloo M., Kiekens L., Velghe G., Camerlynck R. (1982). Chemical Analysis of Plants and Soils.

[52] Pregl F. (1945). Quantitative Organic Micro Analysis.

[53] Kacar B., Inal A. (2008). Plant analysis. Nobel publication No: 1241. Appl. Sci..

[54] Stewart J.M., Guinn G. (1969). Chilling injury and changes in adenosine triphosphate of cotton seedlings. Plant Physiol..

[55] Yan F., Wei H., Ding Y., Li W., Chen L., Ding C., Tang S., Jiang Y., Liu Z., Li G. (2021). Melatonin enhances Na^+^/K^+^ homeostasis in rice seedlings under salt stress through increasing the root H^+^-pump activity and Na^+^/K^+^ transporters sensitivity to ROS/RNS. Environ. Exp. Bot..

[56] Wang Y., Sze H. (1985). Similarities and differences between the tonoplast-type and the mitochondrial H+-ATPases of oat roots. J. Biol. Chem..

[57] Ohnishi T., Gall R.S., Mayer M.L. (1975). An improved assay of inorganic phosphate in the presence of extralabile phosphate compounds: Application to the ATPase assay in the presence of phosphocreatine. Anal. Biochem..

[58] Nawaz M.A., Jiao Y., Chen C., Shireen F., Zheng Z., Imtia M., Bie Z., Huang Y. (2018). Melatonin pretreatment improves vanadium stress tolerance of watermelon on seedlings by reducing vanadium concentration in the leaves and regulating melatonin biosynthesis and antioxidant-related gene expression. J. Plant Physiol..

[59] Gao W., Feng Z., Bai Q., He J., Wang Y. (2019). Melatonin-mediated regulation of growth and antioxidant capacity in salt-tolerant naked oat under salt stress. Int. J. Mol. Sci..

[60] Ramel F., Sulmon C., Bogard M., Couée I., Gouesbet G. (2009). Differential patterns of reactive oxygen species and antioxidative mechanisms during atrazine injury and sucrose-induced tolerance in *Arabidopsis thaliana* plantlets. BMC Plant Biol..

[61] Hossain M.A., Nakano Y., Asada K. (1984). Monodehydroascorbate reductase in spinach chloroplasts and its participation in regeneration of ascorbate for scavenging hydrogen peroxide. Plant Cell Physiol..

[62] Doulis A.G., Debian N., Kingston-Smith A.H., Foyer C.H. (1997). Differential localization of antioxidants in maize. Plant Physiol..

[63] Zhu H., Cao Z., Zhang L., Trush M.A., Li Y. (2007). Glutathione and glutathione-linked enzymes in normal human aortic smooth muscle cells: Chemical inducibility and protection against reactive oxygen and nitrogen species-induced injury. Mol. Cell. Biochem..

[64] Hernandez M., Fernandez-Garcia N., Diaz-Vivancos P., Olmos E. (2010). A different role for hydrogen peroxide and the antioxidative system under short and long salt stress in *Brassica oleracea* roots. J. Exp. Bot..

[65] Enyedi A.J., Yalpani N., Silverman P., Raskin I. (1992). Localization, conjugation, and function of salicylic acid in tobacco during the hypersensitive reaction to tobacco mosaic virus. Proc. Natl. Acad. Sci. USA.

[66] Seskar M., Shulaev V., Raskin I. (1998). Endogenous methyl salicylate in pathogen-inoculated tobacco plants. Plant Physiol..

[67] Naka Y., Watanabe K., Sagor G.H.M., Niitsu M., Pillai M.A., Kusano T., Takahashi Y. (2010). Quantitative analysis of plant polyamines including thermospermine during growth and salinity stress. Plant Physiol. Biochem..

